# Correlation analysis between *CARMEN* variants and alcohol-induced osteonecrosis of the femoral head in the Chinese population

**DOI:** 10.1186/s12891-020-03553-2

**Published:** 2020-08-15

**Authors:** Yongchang Guo, Yuju Cao, Shunguo Gong, Sumei Zhang, Fengzhi Hou, Xinjie Zhang, Jiangeng Hu, Zhimin Yang, Juanjuan Yi, Dan Luo, Xifeng Chen, Jingbo Song

**Affiliations:** Department of Orthopedics, Zhengzhou Traditional Chinese Hospital of Orthopaedics, #1226 East Hanghang Road, Zhengzhou, 450000 Henan China

**Keywords:** Osteonecrosis of the femoral head, Chinese, *Cardiac mesoderm enhancer-associated non-coding RNA*, Polymorphism

## Abstract

**Background:**

Osteonecrosis of the femoral head (ONFH) is a complicated disease associated with trauma, hormone abuse and excessive alcohol consumption. Polymorphisms of long non-coding RNAs have been also linked with the development of ONFH. Our research aimed to explore the relationship between *CARMEN* (*Cardiac Mesoderm Enhancer-Associated Non-Coding RNA*) variants and ONFH risk.

**Methods:**

Our study used Agena MassARRAY Assay to genotype 6 selected single nucleotide polymorphisms (SNPs) in 731 participants (308 alcohol-induced ONFH patients and 423 controls). We used odds ratios (ORs) and 95% confidence intervals (CIs) to calculate the effect of gene polymorphisms on the occurrence of alcohol-induced ONFH by logistic regression analysis and haplotype analysis.

**Results:**

Our overall analysis illustrated that rs13177623 and rs12654195 had an association with a reduced risk of ONFH after adjustment for age and gender. We also found that rs13177623, rs12654195 and rs11168100 were associated with a decreased susceptibility to alcohol-induced ONFH in people ≤45 years. In addition, the necrotic sites stratification analysis showed that rs12654195 was only found to be related to alcohol-induced ONFH risk in the recessive model. In patients with different clinical stages, rs353300 was observed to be associated with a higher incidence of ONFH. Individuals with different genotypes of rs13177623, rs12654195 and rs11168100 had significantly different clinical parameters (cholinesterase, globulin, percentage of neutrophils and the absolute value of lymphocytes).

**Conclusions:**

Our data provided new light on the association between *CARMEN* polymorphisms and alcohol-induced ONFH risk in the Chinese Han population.

## Background

Osteonecrosis of the femoral head (ONFH) is a devastating orthopedic disease, which is characterized by bone cell death due to the damage of microvascular circulation, which is considered to be the result of mechanical vascular disruption, intravascular occlusion and extravascular compression [1, 2]. ONFH usually occurs in people aged 30–50 years and this refractory disease has a high disability rate [3]. Over the past few decades, the incidence rate of ONFH has been increasing worldwide. Every year, 20,000 people in the United States are diagnosed with ONFH [4], and 2200 people in Japan are diagnosed as ONFH [5]. It is estimated that there are 8.12 million ONFH cases in Chinese people aged 15 years and over [6]. Increasing evidence showed that ONFH is a complex disease associated with many factors, such as trauma [7], genetic factors [8], high-dose corticosteroid use [9] and excessive alcohol consumption. Excessive alcohol intake and steroids are considered to be the main environmental risk factors [6, 10]. According to statistics in China, 30.7% of ONFH cases are caused by alcohol [11]. Thereinto, genetic polymorphisms of some genes played critical roles in the occurrence of alcohol**-**induced ONFH [12–17], such as MMP20, RETN, ApoB, ApoA1, NOS3.

Recently, long non-coding RNAs (lncRNAs), a set of transcribed RNA molecules with a length of more than 200 nucleotides, do not have the ability to encode protein. But these transcripts are able to modulate the target gene expression with the cis-trans regulation. They are involved in the development of many diseases by regulating the mechanisms related to epigenetic modification, transcription and post-processing. As some reports went, not only were they involved in cell proliferation and cell differentiation, but also they were related to tumorigenesis [18, 19]. In addition, lncRNA, an important regulatory medium, has been reported to be of cardiac lineage specificity in the development process and to have special cellular functions in maintaining cardiac integrity [20, 21].

*CARMEN* (*Cardiac Mesoderm Enhancer-Associated Non-Coding RNA*) is also known as *CLAP*, *MIR143HG*. *Carmen* is reported to be highly conserved in mice, and is an important regulatory factor for cardiovascular differentiation. In human, it is found to be active in the heart. And Du et al. found that *MIR143HG*, as a pathogenic factor, could control the level of RBM24(RNA binding motif protein 24) in Hirschsprung disease (HSCR) positively through the marine spreading of miR-143 [22]. Conversely, RBM24 reduced MIR143HG expression by reducing its stability and promoting the synthesis of miR-143. However, the function of MIR143HG in the development of ONFH hasn’t been reported until now.

Here, we did a case-control study to investigate the association between *CARMEN* variants and ONFH risk in the Chinese Han population, which contributes to knowing about the role of *CARMEN* in the development of ONFH and is helpful for identifying patients with high-risk alcoholic ONFH.

## Methods

### Subjects

Totally, 731 male participants (308 alcohol-induced ONFH patients and 423 controls) were recruited by the Zhengzhou Traditional Chinese Hospital of Orthopaedics. Our cases meet the following criteria: 1) The patient’s alcohol intake have more than 400 mL/week [23] (320 g/week, any type of alcoholic beverage) for more than 6 months; 2) ONFH was diagnosed within one year after drinking; 3) Patients had no hyperlipidemia, rheumatoid arthritis, spinal cord cavitation, osteoporosis, decompression sickness, cardiovascular disease and human immunodeficiency virus infection, and no history of steroid use or smoking; 4) The diagnosis of alcohol-induced ONFH was assessed by X-ray, computed tomography(CT), nuclear magnetic resonance imaging (MRI). In the process, ONFH diagnosis was evaluated by Classifcation system [24] originally proposed by Ficat and Arlet. The selection criteria of all healthy people were: 1) Members of the control group had drinking habits and alcohol intake is greater than 400 mL per week (320 g/week, any type of alcoholic beverage) for more than 6 months; 2) They had no history of traumatic disease (ONFH, hyperlipidemia, rheumatoid arthritis, spinal cord cavitation, osteoporosis, decompression sickness, cardiovascular disease, steroid use, smoking, etc).

### DNA extraction, SNP selection and genotyping

At least 10 h after fasting, 5 mL of venous peripheral blood samples of participants were collected by professional medical personnel and stored in an EDTA anticoagulation tube and − 80 °C refrigerator. We extracted genomic DNA by using the GoldMag-Mini Whole Blood Genomic DNA Purification Kit (GoldMag Co. Ltd., Xi’an, China). A NanoDrop 2000C spectrophotometer (Thermo Scientifc, Waltham, MA, USA) was applied to detect the DNA concentration and purity. Our present study selected 6 variants located in *CARMEN* selected from the 1000 Genome Project (https://www.internationalgenome.org/) with minor allele frequencies (MAFs) > 5% in the global population [25]. Amplification and extension of primers were designed using the Agena MassARRAY Assay Design 3.0 software (Agena, Inc., San Diego, CA, USA). Agena MassARRAY RS1000 (Agena, Inc., San Diego, CA, USA) was used to perform SNP genotyping according to the standard process [16, 25]. In the end, we completed the data processing with Agena Bioscience TYPER software, version 4.0 [17].

### Statistical analysis

Age and sex differences between cases and controls were assessed by Student’s t-test and Pearson′s chi-square, respectively. In addition, we did the genotype distribution of locus in the control group, in order to further explain the good representativeness of the study population. PLINK 1.07 software (Harvard, Boston, MA, USA) [26] was utilized to calculate the association between SNPs and alcohol-induced ONFH risk by logistic regression analysis with ORs and 95%CI. The version 4.2 of Haploview software (Harvard, Boston, MA, USA) was used to calculate the degree of linkage among these SNPs provided by a linkage disequilibrium (LD) map [27]. *p*-value was two-tailed and *p*-value <0.05 was considered statistically significant.

## Results

### Basic information of study subjects

The information of subjects was listed in Table 1. Of population recruited from Department of Orthopedics of Zhengzhou Chinese Hospital, the mean age of 308 cases and 423 controls were 43.47 ± 11.303 years and 42.52 ± 13.135 years, respectively. No significant difference was found in age and gender between the two groups. In addition, clinical information analysis (hip lesions, clinical stages and course) was also included in the study.

**Table 1:** The basic information of subjects

Characteristics	Cases N(%)	Controls N(%)	*p*-value
Number	308	423	
Age, year (mean ± SD)	43.47 ± 11.303	42.52 ± 13.135	0.396
> 45	133 (43%)	198 (47%)	
≤ 45	175 (57%)	225 (53%)	
Hip lesions			
Unilateral	66 (21%)		
Bilateral	242 (79%)		
Clinical stages			
III/IV	218 (71%)		
I/II	90 (29%)		

*ONFH* Osteonecrosis of the femoral head; *TC* Total cholesterol; *TG* Triglycerides; *LDL-C* Low-density lipoprotein-cholesterol; *HDL-C* High-density lipoprotein-cholesterol

### Basic information of selected SNP

The information of selected SNPs located in *CARMEN* was shown in Table 2. In Table 2, we listed the chromosome position, specific locations, minor/major alleles, minor allele frequency in cases and controls, HWE (Hardy-Weinberg equilibrium) and allele model. Every polymorphism was in accordance with HWE. In the allele model, six variants (rs13177623, rs12654195, rs11168100, rs353303, rs353300 and rs353299) did not appear to be associated with alcohol-induced ONFH.

**Table 2:** The basic information of selected SNPs located in *CARMEN*

SNP ID	Gene	Chromosome position	Role	Alleles	MAF		HWE- *p*-value	OR (95% CI)	*p*-value
				(minor/major)	Case	Control			
rs13177623	*CARMEN*	chr5: 149408144	Intron	A/G	0.271	0.310	0.734	0.83 (0.66–1.04)	0.110
rs12654195	*CARMEN*	chr5: 149409947	Intron	G/T	0.312	0.354	0.087	0.83 (0.66–1.03)	0.090
rs11168100	*CARMEN*	chr5: 149413801	Intron	A/T	0.307	0.342	0.449	0.85 (0.68–1.07)	0.162
rs353303	*CARMEN*	chr5: 149419554	Intron	C/T	0.397	0.400	0.543	0.99 (0.80–1.22)	0.906
rs353300	*CARMEN*	chr5: 149421006	Intron	A/G	0.516	0.480	0.206	1.16 (0.94–1.42)	0.170
rs353299	*CARMEN*	chr5: 149421538	Intron	A/G	0.154	0.149	0.177	1.04 (0.78–1.39)	0.781

*95%CI* 95% Confidence interval; *HWE* Hardy-Weinberg equilibrium; *MAF* Minor allele frequency; *OR* Odds ratio; *SNP* Single-nucleotide polymorphism

*p*-value: Calculated by Pearson χ^2^ test

### Relationship between *CARMEN* variants and alcohol-induced ONFH risk

Four genetic models (codominant, dominant, recessive and log-additive) were also used to analyze the relationship between six *CARMEN* variants and alcohol-induced ONFH risk (Table 3). In the codominant model, individuals with rs13177623 G/G genotype had a smaller possibility of ONFH compared to the AA genotype (adjusted OR = 0.52, 95%CI: 0.28–0.94, *p* = 0.031). The recessive model also illustrated that rs13177623 G/G conferred a decreased susceptibility to alcohol-induced ONFH risk in comparison with A/A-A/G (adjusted OR = 0.53, 95%CI: 0.30–0.95, *p* = 0.033). Rs12654195 was also found to be associated with a decreased susceptibility of alcohol-induced ONFH in the codominant (adjusted OR = 0.53, 95%CI: 0.32–0.90, *p* = 0.017) and recessive (adjusted OR = 0.53, 95%CI: 0.32–0.86, *p* = 0.011) models.

**Table 3:** Association between *CARMEN* variants and ONFH risk

SNP-ID	Model	Genotype	Frequency		Without adjustment		With adjustment	
			Case	Control	OR (95% CI)	*p*-value	OR (95% CI)	*p*-value
rs13177623	codominant	A/A	17	42	1		1	
		A/G	133	178	0.96 (0.71–1.30)	0.794	0.95 (0.70–1.29)	0.726
		G/G	158	203	0.52 (0.29–0.95)	0.377	0.52 (0.28–0.94)	**0.031**
	dominant	A/A	17	42	1		1	
		A/G-G/G	291	381	0.88 (0.65–1.18)	0.341	0.86 (0.64–1.16)	0.332
	recessive	A/A-A/G	150	220	1		1	
		G/G	158	203	0.53 (0.30–0.95)	**0.033**	0.53 (0.30–0.95)	**0.033**
	log-additive	–	–	–	0.82 (0.65–1.04)	0.106	0.82 (0.65–1.03)	0.091
rs12654195	codominant	G/G	25	60	1		1	
		G/T	142	174	1.05 (0.77–1.43)	0.771	1.04 (0.76–1.42)	0.831
		T/T	141	181	0.53 (0.32–0.90)	**0.017**	0.53 (0.32–0.90)	**0.017**
	dominant	G/G	25	60	1		1	
		G/T-T/T	283	355	0.92 (0.68–1.23)	0.563	0.91 (0.67–1.22)	0.517
	recessive	G/G-G/T	167	234	1		1	
		T/T	141	181	0.52 (0.32–0.85)	**0.010**	0.53 (0.32–0.86)	**0.011**
	log-additive	–	–	–	0.83 (0.66–1.03)	0.094	0.82 (0.66–1.03)	0.087

*95%CI* 95% Confidence interval; *OR* Odds ratio; *SNP* Single-nucleotide polymorphism

*p*-value: Calculated by Pearson χ^2^ test

Bold type indicates statistical significance (*p* < 0.05)

### Stratification analysis of the association between *CARMEN* variants and alcohol-induced ONFH risk

We further assessed the relationship between *CARMEN* variants and alcohol-induced ONFH risk in > 45 years groups and ≤ 45 years groups (Table 4). But these sites were only found to be associated with alcohol-induced ONFH in people younger than 45 years. Rs12654195 T was correlated with a reduced risk of alcohol-induced ONFH compared to the allele G (adjusted OR = 0.69, 95%CI: 0.51–0.93, *p* = 0.015). There was non-significance between rs13177623 G, rs11168100 T and alcohol-induced ONFH susceptibility in contrast with wide type allele. In the codominant, recessive and log-additive models, rs13177623 conferred a decreased susceptibility to alcohol-induced ONFH (adjusted OR = 0.39, 95%CI: 0.18–0.87, *p* = 0.022; adjusted OR = 0.44, 95%CI: 0.20–0.96, *p* = 0.038; adjusted OR = 0.69, 95%CI: 0.50–0.95, *p* = 0.021). Also, rs12654195 was associated with the risk of alcohol-induced ONFH in four models (*p* = 0.008, *p* = 0.033, *p* = 0.019, *p* = 0.008). Whereas, rs11168100 was only related to alcohol-induced ONFH risk in the log-additive model (adjusted OR = 0.73, 95%CI: 0.54–0.99, *p* = 0.045).

**Table 4:** Association between *CARMEN* variants and ONFH risk stratified by age

SNP	Model	Genotype	>45ys		≤45ys	
			OR(95%CI)	*p-*value	OR(95%CI)	*p*-value
rs13177623	Allele	A	1		1	
		G	0.97 (0.69–1.36)	0.868	0.73 (0.53–1.00)	0.047
	codominant	A/A	1		1	
		A/G	1.15 (0.73–1.81)	0.557	0.77 (0.50–1.19)	0.239
		G/G	0.71 (0.27–1.84)	0.475	0.39 (0.18–0.87)	**0.022**
	dominant	A/A	1		1	
		A/G-G/G	1.08 (0.7–1.68)	0.726	0.69 (0.45–1.03)	0.072
	recessive	A/A-A/G	1		1	
		G/G	0.66 (0.26–1.66)	0.377	0.44 (0.20–0.96)	**0.038**
	log-additive	–	0.99 (0.69–1.41)	0.936	0.69 (0.50–0.95)	**0.021**
rs12654195	Allele	G	1		1	
		T	1.03 (0.74–1.44)	0.868	0.69 (0.51–0.93)	**0.015**
	codominant	G/G	1		1	
		G/T	1.47 (0.92–2.34)	0.108	0.74 (0.47–1.15)	0.176
		T/T	0.65 (0.27–1.59)	0.349	0.41 (0.21–0.79)	**0.008**
	dominant	G/G	1		1	
		G/T-T/T	1.31 (0.83–2.05)	0.243	0.64 (0.42–0.97)	**0.033**
	recessive	G/G-G/T	1		1	
		T/T	0.53 (0.23–1.25)	0.147	0.47 (0.25–0.88)	**0.019**
	log-additive	–	1.05 (0.74–1.49)	0.796	0.67 (0.49–0.90)	**0.008**
rs11168100	Allele	A	1		1	
		T	1.00 (0.72–1.39)	1.000	0.75 (0.55–1.01)	0.061
	codominant	A/A	1		1	
		A/T	1.39 (0.88–2.21)	0.160	0.71 (0.46–1.11)	0.132
		T/T	0.63 (0.26–1.52)	0.302	0.55 (0.28–1.08)	0.084
	dominant	A/A	1		1	
		A/T-T/T	1.24 (0.8–1.94)	0.340	0.67 (0.45–1.02)	0.059
	recessive	A/A-A/T	1		1	
		T/T	0.53 (0.23–1.23)	0.136	0.64 (0.34–1.23)	0.181
	log-additive	–	1.01 (0.72–1.43)	0.946	0.73 (0.54–0.99)	**0.045**

*95%CI* 95% Confidence interval; *OR* Odds ratio; *SNP* Single-nucleotide polymorphism

*p*-value: Calculated by Pearson χ^2^ test

Bold type indicates statistical significance (*p* < 0.05)

In addition, we did the necrotic sites stratification analysis to evaluate the association between *CARMEN* variants and alcohol-induced ONFH risk (bilateral ONFH patients vs controls) shown in Table 5. Rs12654195 was only found to be correlated with alcohol-induced ONFH risk in the recessive model (adjusted OR = 0.60, 95%CI: 0.35–0.99, *p* = 0.049).

**Table 5:** Association between *CARMEN* variants and ONFH risk stratified by necrotic sites

SNP	Model	Genotype	Frequency		Without adjustment		With adjustment	
			Case	Control	OR(95%CI)	*p-*value	OR(95%CI)	*p*-value
rs12654195	codominant	G/G	22	60	1		1	
		G/T	111	174	1.06 (0.76–1.48)	0.737	1.05 (0.75–1.47)	0.780
		T/T	109	181	0.61 (0.35–1.05)	0.073	0.61 (0.35–1.05)	0.074
	dominant	G/G	22	60	1		1	
		G/T-T/T	220	355	0.94 (0.69–1.30)	0.722	0.94 (0.68–1.29)	0.688
	recessive	G/G-G/T	133	234	1		1	
		T/T	109	181	0.59 (0.35–0.99)	**0.046**	0.60 (0.35–0.99)	**0.049**
	log-additive	–	142	174	0.86 (0.68–1.09)	0.219	0.86 (0.68–1.09)	0.211

*95%CI* 95% Confidence interval; *OR* Odds ratio; *SNP* Single-nucleotide polymorphism

*p*-value: Calculated by Pearson χ^2^ test

Bold type indicates statistical significance (*p* < 0.05)

### Analysis of the association between *CARMEN* variants and alcohol-induced ONFH risk in patients with different clinical stages and clinical parameters

We also investigated the association between *CARMEN* variants and alcohol-induced ONFH risk in patients with different clinical stages and clinical parameters (Supplementary Table 2 and Supplementary Table 3). In the Supplementary Table 2, stage III and IV individuals were used as the case group, while stage I and II individuals as the control group. The results showed that subjects with rs353300 TC genotype (adjusted OR = 1.83, 95%CI: 1.01–3.32, *p* = 0.046) or TT genotype (adjusted OR = 2.27, 95%CI: 1.12–4.57, *p* = 0.002) had a higher incidence of ONFH compared with patients with CC genotype, up to 1.83-fold and 2.27-fold, respectively. When comparing to the CC genotype in the dominant model, patients with rs353300 T/C-T/T genotype had a higher likelihood of developing into ONFH (adjusted OR = 1.97, 95%CI: 1.13–3.44, *p* = 0.017). The log-additive model also explained that rs353300 was correlated with an increased risk of alcohol-induced ONFH by 1.52-fold (adjusted OR = 1.52, 95%CI: 1.06–2.17, *p* = 0.022).

Moreover, we analyzed the relationship between genotypes of different loci and clinical parameters (cholinesterase, globulin, percentage of neutrophils and absolute value of lymphocytes) in the Supplementary Table 3. We found that the absolute value of lymphocyte (LYMPH) were significantly different among rs13177623 carriers of different genotypes (*p* = 0.016). The content of CHE, GLO and LYMPH were also significantly different among rs12654195 carriers with different genotypes (*p* = 0.027, *p* = 0.022, *p* = 0.013). But, there was no difference in NEUT content among different genotypes of rs12654195 carriers. To our surprise, carriers of different genotypes of rs11168100 had obvious difference in the content of CHE, GLO, NEUT and LYMPH (*p* = 0.010, *p* = 0.011, *p* = 0.048, *p* = 0.014).

### LD and haplotype analysis

**Fig. 1:**
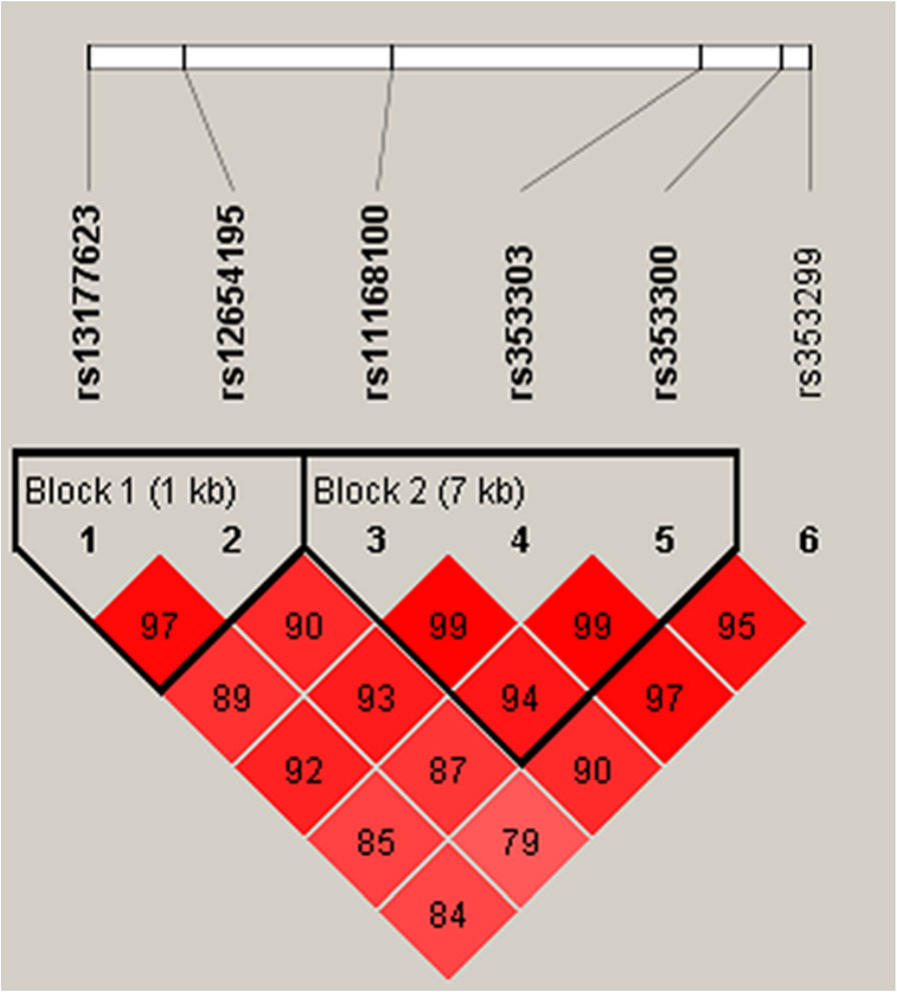
Linkage disequilibrium (LD) analysis of SNPs in *CARMEN*. The LD value is determined by *r*^*2*^ > 0.8 analyzed by Haploview software 4.2. The number in the diamonds is the LOD score of r^2^. Standard color schemes indicated different levels of LD. Bright red: LOD > 2, D’ = 1

Among the six variants (rs13177623, rs12654195, rs11168100, rs353303, rs353300 and rs353299), we completed the LD analysis (Fig. 1 and Supplementary Table 4). There was a 1 kb LD block1 between rs13177623 and rs12654195, and rs11168100, rs353303, rs353300 formed a 7 kb LD block2. Totally, AAC haplotype was associated with an increased alcohol-induced ONFH risk by 1.62-fold (adjusted OR = 1.62, 95%CI: 1.14–2.30, *p* = 0.007). After age stratification analysis, five haplotypes (AG, GT, TA, AA and TC) were related to a decreased alcohol-induced ONFH risk in ≤45 years patients. Haplotype AA showed an increased alcohol-induced ONFH risk in ≤45 years patients (adjusted OR = 1.64, 95%CI: 1.20–2.25, *p* = 0.002). And after necrotic sites stratification analysis, AAC haplotype was also found to be associated with an increased alcohol-induced ONFH risk in patients with bilateral necrotic sites (adjusted OR = 1.72, 95%CI: 1.19–2.49, *p* = 0.004).

## Discussion

Our case-control study illustrated that *CARMEN* variants were related to the risk of ONFH. Rs13177623, rs12654195 and rs11168100 were associated with alcohol-induced ONFH in people younger than 45 years. Rs12654195 was only found to be related to alcohol-induced ONFH risk after the necrotic sites stratification analysis. In patients with different clinical stages, rs353300 was observed to be associated with a higher incidence of ONFH. While, individuals with different genotypes of rs13177623, rs12654195 and rs11168100 had significantly different levels of cholinesterase, globulin, percentage of neutrophils, and the absolute value of lymphocytes.

*CARMEN* (also named as MiR143HG), was differentially expressed in cardiac progenitor cells and proliferating cells in cardiovascular pedigree, which was first described by Ouzain et al. [28]. It belongs to the intergenic lncRNA group of ncRNAs and has multiple exon splicing variants. And a previous analysis of promoter specific histone modification and poly (a) signal frequency indicated that the boundaries of two *CARMEN* transcripts were clear [29]. Chromatin state analysis showed that the expression of MiR143HG isoforms was up-regulated in the process of cardiogenic differentiation between mesoderm and cardiac precursor cell (CPC) [30]. Consistent with this observation, Ounzain et al. found that it was not only expressed in the process of myocardial differentiation, but also in the adult mouse and human heart [31]. MiR143HG knockdown by shRNA or GapmeR can influence the significant reduction of cardiac differentiation markers to impair CPC differentiation [28]. The above research showed that MiR143HG played a role in heart disease, which may affect the occurrence and development of the disease by affecting blood circulation. Our study was the first to show that MiR143HG was related to the risk of necrosis of the femoral head, and the cause of necrosis of the femoral head is the obstruction of microvascular circulation. Although we did not find a link between *CARMEN* variants and alcohol-induced ONFH risk in people over 45 years old, *CARMEN* variants were found to be related to a reduced risk of alcohol-induced ONFH in people less than 45 years, which may be related to their relatively smooth blood circulation. Therefore, we speculate that MiR143HG had a certain role in the microvascular circulation of necrosis of the femoral head.

In conclusion, we found that *CARMEN* variants were associated with alcohol-induced ONFH risk. It proved that *CARMEN* may play a crucial role in the occurrence of ONFH. In spite of some limitations, our results are helpful for the follow-up study of alcohol-induced osteonecrosis of the femoral head. In future, we will use larger samples and rat models of alcoholic osteonecrosis to verify the results.

## Conclusion

In conclusion, the study provides new light on the correlation of *CARMEN* polymorphisms with alcohol-induced ONFH risk in the Chinese Han population.

## Supplementary information

**Additional file 1 Supplementary Table 1** The primers information of selected SNPs

**Additional file 2 Supplementary Table 2** Association between *CARMEN* variants and ONFH risk in patients with different clinical stages

**Additional file 3 Supplementary Table 3** The relationship between genotypes of different loci and clinical parameters

**Additional file 4 Supplementary Table 4** *CARMEN* haplotypes frequencies associated with ONFH risk

## Data Availability

The datasets used and/or analysed during the current study are available from the corresponding author on reasonable request.

## Abbreviations

SNPs: Single nucleotide polymorphisms
OR: Odds ratio
95%CI: 95% Confidence interval
C: ARMEN Cardiac mesoderm enhancer-associated non-coding RNA
LD: Linkage disequilibrium
HWE: Hardy-Weinberg equilibrium
MAF: Minor allele frequency

## References

[1] Mouzas OD, Zibis AH, Bonotis KS, Katsimagklis CD, Hadjigeorgiou GM, Papaliaga MN, Dimitroulias AP, Malizos KN (2014). Psychological distress, personality traits and functional disability in patients with osteonecrosis of the femoral head. J Clin Med Res.

[2] Shah KN, Racine J, Jones LC, Aaron RK (2015). Pathophysiology and risk factors for osteonecrosis. Curr Rev Musculoskelet Med.

[3] Zalavras CG, Lieberman JR (2014). Osteonecrosis of the femoral head: evaluation and treatment. J Am Acad Orthop Surg.

[4] Adesina OO, Brunson A, Keegan THM, Wun T (2016). Osteonecrosis of the femoral head in sickle cell disease: prevalence, comorbidities and surgical outcomes in California. Blood Adv.

[5] Kubo T, Ueshima K, Saito M, Ishida M, Arai Y, Fujiwara H (2016). Clinical and basic research on steroid-induced osteonecrosis of the femoral head in Japan. J Orthop Sci.

[6] Zhao D, Yu M, Hu K, Wang W, Yang L, Wang B, Gao X, Guo Y, Xu Y, Wei Y (2015). Prevalence of nontraumatic osteonecrosis of the femoral head and its associated risk factors in the Chinese population: results from a nationally representative survey. Chin Med J.

[7] Choi HR, Steinberg ME, Cheng EY (2015). Osteonecrosis of the femoral head: diagnosis and classification systems. Curr Rev Musculoskelet Med.

[8] Wang L, Pan H, Zhu ZA (2014). A genetic pedigree analysis to identify gene mutations involved in femoral head necrosis. Mol Med Rep.

[9] Xue Y, Zhao Z, Hong D, Zhang H, Chen H, Fan S (2014). MDR1 gene polymorphisms are associated with glucocorticoid-induced avascular necrosis of the femoral head in a Chinese population. Genet Test Mol Biomarkers.

[10] Tsai S, Wu P, Chen C, Chiang C, Huang C, Chen T, Liu C, Chen W (2016). Etiologies and outcome of osteonecrosis of the femoral head: etiology and outcome study in a Taiwan population. Journal of The Chinese Medical Association.

[11] Cui L, Zhuang Q, Lin J, Jin J, Zhang K, Cao L, Lin J, Yan S, Guo W, He W (2016). Multicentric epidemiologic study on six thousand three hundred and ninety five cases of femoral head osteonecrosis in China. Int Orthop.

[12] An F, Du J, Wang J, Zhao L, Ma C, Zhao J, Wang J (2019). MMP20 single-nucleotide polymorphisms correlate with susceptibility to alcohol-induced osteonecrosis of the femoral head in Chinese males. Med Sci Monit.

[13] Liu C, An F, Cao Y, Wang J, Tian Y, Wu H, Wang J (2019). Significant association between RETN genetic polymorphisms and alcohol-induced osteonecrosis of femoral head. Mol Genet Genomic Med.

[14] Wang Y, Cao Y, Li Y, Guo Y, Wang Q, Yang M, Zhang N, Jin T, Wang J (2015). Genetic association of the ApoB and ApoA1 gene polymorphisms with the risk for alcohol-induced osteonecrosis of femoral head. Int J Clin Exp Pathol.

[15] Wang Y, Yang X, Shi J, Zhao Y, Pan L, Zhou J, Wang G, Wang J (2017). Combination analysis of NOS3, ABCB1 and IL23R polymorphisms with alcohol-induced osteonecrosis of the femoral head risk in Chinese males. Oncotarget.

[16] Chen J, Liu W, Cao Y, Zhang X, Guo Y, Zhu Y, Li J, Du J, Jin T, Wang G (2017). MMP-3 and MMP-8 single-nucleotide polymorphisms are related to alcohol-induced osteonecrosis of the femoral head in Chinese males. Oncotarget.

[17] Li Y, Wang Y, Guo Y, Wang Q, Ouyang Y, Cao Y, Jin T, Wang J (2016). OPG and RANKL polymorphisms are associated with alcohol-induced osteonecrosis of the femoral head in the north area of China population in men. Medicine.

[18] Gibb EA, Brown CJ, Lam WL (2011). The functional role of long non-coding RNA in human carcinomas. Mol Cancer.

[19] Su SC, Reiter RJ, Hsiao HY, Chung WH, Yang S (2018). Functional interaction between melatonin signaling and noncoding RNAs. Trends Endocrinol Metab.

[20] Klattenhoff CA, Scheuermann JC, Surface LE, Bradley RK, Fields PA, Steinhauser ML, Ding H, Butty VL, Torrey L, Haas S (2013). Braveheart, a long noncoding RNA required for cardiovascular lineage commitment. Cell.

[21] Ounzain S, Micheletti R, Beckmann T, Schroen B, Alexanian M, Pezzuto I, Crippa S, Nemir M, Sarre A, Johnson R (2014). Genome-wide profiling of the cardiac transcriptome after myocardial infarction identifies novel heart-specific long non-coding RNAs. Eur Heart J.

[22] Du C, Shen Z, Zang R, Xie H, Li H, Chen P, Hang B, Xu X, Tang W, Xia Y (2016). Negative feedback circuitry between MIR143HG and RBM24 in Hirschsprung disease. Biochim Biophys Acta.

[23] Matsuo K, HIROHATA T, SUGIOKA Y, IKEDA M, FUKUDA A (1988). Influence of alcohol intake, cigarette smoking, and occupational status on idiopathic osteonecrosis of the femoral head. Clin Orthop Relat Res.

[24] Totty WG, Murphy WA, Ganz W, Kumar B, Daum WJ, Siegel BA (1984). Magnetic resonance imaging of the normal and ischemic femoral head. Am J Roentgenol.

[25] Du J, Jin T, Cao Y, Chen J, Guo Y, Sun M, Li J, Zhang X, Wang G, Wang J (2016). Association between genetic polymorphisms of MMP8 and the risk of steroid-induced osteonecrosis of the femoral head in the population of northern China. Medicine.

[26] Purcell S, Neale BM, Toddbrown K, Thomas L, Ferreira MAR, Bender D, Maller J, Sklar P, De Bakker PIW, Daly MJ (2007). PLINK: a tool set for whole-genome association and population-based linkage analyses. Am J Hum Genet.

[27] Yu Y, Xie Z, Wang J, Chen C, Du S, Chen P, Li B, Jin T, Zhao H (2016). Single-nucleotide polymorphisms of MMP2 in MMP/TIMP pathways associated with the risk of alcohol-induced osteonecrosis of the femoral head in Chinese males: a case-control study. Medicine (Baltimore).

[28] Ounzain S, Micheletti R, Arnan C, Plaisance I, Cecchi D, Schroen B, Reverter F, Alexanian M, Gonzales C, Ng SY (2015). CARMEN, a human super enhancer-associated long noncoding RNA controlling cardiac specification, differentiation and homeostasis. J Mol Cell Cardiol.

[29] Derrien T, Johnson R, Bussotti G, Tanzer A, Djebali S, Tilgner H, Guernec G, Martin D, Merkel A, Knowles DG (2012). The GENCODE v7 catalog of human long noncoding RNAs: analysis of their gene structure, evolution, and expression. Genome Res.

[30] Boucher JM, Peterson SM, Urs S, Zhang C, Liaw L (2011). The miR-143/145 cluster is a novel transcriptional target of Jagged-1/notch signaling in vascular smooth muscle cells. J Biol Chem.

[31] Ounzain S, Micheletti R, Beckmann T, Schroen B, Alexanian M, Pezzuto I, Crippa S, Nemir M, Sarre A, Johnson R (2015). Genome-wide profiling of the cardiac transcriptome after myocardial infarction identifies novel heart-specific long non-coding RNAs. Eur Heart J.

