# Supplementary material for: Correlation analysis between CARMEN variants and alcohol-induced osteonecrosis of the femoral head in the Chinese population
Source: BMC Musculoskelet Disord. 2020 Aug 15;21:547. doi: 10.1186/s12891-020-03553-2 (PMC7429464; doi:10.1186/s12891-020-03553-2)
Supplement: Supplementary file 1 — Additional file 1 Supplementary Table 1 The primers information of selected SNPs [file 12891_2020_3553_MOESM1_ESM.docx]

Supplementary Table 1 The primers information of selected SNPs

| SNP_ID | 1st-PCR primer | 2nd-PCR primer | UEP SEQ | | Direction | |  |
| --- | --- | --- | --- | --- | --- | --- | --- |
| rs13177623 | ACGTTGGATGTGGAAGTAGGTAAGGGATGG | ACGTTGGATGTCCCATCACAAGCCTCCTG | AAGCCTCCTGCATTCCT | | R | |  |
| rs12654195 | ACGTTGGATGAAATGAGGTCTCCCTCTGTC | ACGTTGGATGGAAGCAGAGGTTGCAGTCAA | tcctcGGTTGCAGTCAATTGAGAAG | | R | |  |
| rs11168100 | ACGTTGGATGTTGAGGAGGGTACCAAATGC | ACGTTGGATGTAGTCTGTCCAAAGAGGGTG | GGGATTAGGAAGTACAGACAT | | F | |  |
| rs353303 | ACGTTGGATGCTCATCTGATCCTCAAAGTC | ACGTTGGATGCAATAGGTGTTGCCCTGGAA | ggcGGTGTTGCCCTGGAAAGCAAG | | F | |  |
| rs353300 | ACGTTGGATGCGAATGTGCCTTGAGTTACG | ACGTTGGATGGCTTGTGGCTGTCTTTGACC | cctgaTGTCTTTGACCCCTTCC | | R | |  |
| rs353299 | ACGTTGGATGGCTCACAGTTCTGTCTCCG | ACGTTGGATGCCATGCTGATGTCAGAGAAG | tgcTGTCAGAGAAGCAAGAA | | F | |  |
| SNP, single nucleotide polymorphism; UEP SEQ, Unextended mini-sequencing primer. | | | |  | |  | |
