# Supplementary material for: Correlation analysis between CARMEN variants and alcohol-induced osteonecrosis of the femoral head in the Chinese population
Source: BMC Musculoskelet Disord. 2020 Aug 15;21:547. doi: 10.1186/s12891-020-03553-2 (PMC7429464; doi:10.1186/s12891-020-03553-2)
Supplement: Supplementary file 2 — Additional file 2 Supplementary Table 2 Association between CARMEN variants and ONFH risk in patients with different clinical stages [file 12891_2020_3553_MOESM2_ESM.docx]

Supplementary Table 2 Association between *CARMEN* variants and ONFH risk in patients with different clinical stages.

| SNP | Model | Genotype | Frequency | | Without adjustment | | With adjustment | |
| --- | --- | --- | --- | --- | --- | --- | --- | --- |
|  |  |  | Case | Control | OR(95%CI) | *p-*value | OR(95%CI) | *p*-value |
| rs353300 | Allele | C | 238 | 80 | 1 |  | 1 |  |
|  |  | T | 198 | 100 | 1.50(1.06-2.13) | **0.022** | 0.73(0.53-1.00) | **0.047** |
|  | codominant | C/C | 62 | 19 | 1 |  | 1 |  |
|  |  | T/C | 114 | 42 | 1.87(1.04-3.38) | **0.037** | 1.83(1.01-3.32) | **0.046** |
|  |  | T/T | 42 | 29 | 2.25(1.12-4.53) | **0.023** | 2.27(1.12-4.57) | **0.022** |
|  | dominant | C/C | 62 | 19 | 1 |  | 1 |  |
|  |  | T/C-T/T | 156 | 71 | 1.99(1.14-3.47) | **0.015** | 1.97(1.13-3.44) | **0.017** |
|  | recessive | C/C-T/C | 176 | 61 | 1 |  | 1 |  |
|  |  | T/T | 42 | 29 | 1.49(0.83-2.67) | 0.186 | 1.52(0.84-2.74) | 0.163 |
|  | log-additive | – | – | – | 1.52(1.06-2.17) | **0.022** | 1.52(1.06-2.17) | **0.022** |

95%CI: 95% confidence interval; OR: odds ratio; SNP: single-nucleotide polymorphism.

*p*-value: Calculated by Pearson χ^2^ test.

Bold type indicates statistical significance (*p* < 0.05).
