# Supplementary material for: Correlation analysis between CARMEN variants and alcohol-induced osteonecrosis of the femoral head in the Chinese population
Source: BMC Musculoskelet Disord. 2020 Aug 15;21:547. doi: 10.1186/s12891-020-03553-2 (PMC7429464; doi:10.1186/s12891-020-03553-2)
Supplement: Supplementary file 3 — Additional file 3 Supplementary Table 3 The relationship between genotypes of different loci and clinical parameters [file 12891_2020_3553_MOESM3_ESM.docx]

Supplementary Table 3 The relationship between genotypes of different loci and clinical parameters

| SNP | CHE (mmol/L) | GLO (mmol/L) | NEUT (mmol/L) | LYMPH (mmol/L) |
| --- | --- | --- | --- | --- |
| rs13177623 |  |  |  |  |
| AA | 8904±1559.26 | 23.82±2.22 | 56.86±10.95 | 2.72±0.96 |
| AG | 8068.09±1878.15 | 24.28±4.17 | 60.85±9.48 | 2.17±0.84 |
| GG | 7804.62±1981.53 | 23.13±3.60 | 62.15±8.71 | 2.02±0.70 |
| *p* | 0.214 | 0.136 | 0.173 | **0.016** |
| rs12654195 |  |  |  |  |
| GG | 7707.33±1932.42 | 22.89±3.57 | 62.45±8.53 | 1.98±0.67 |
| GT | 8057.09±1782.73 | 24.5±4.12 | 60.97±9.69 | 2.19±0.85 |
| TT | 9086.31±2267.35 | 23.46±2.67 | 56.51±9.21 | 2.58±0.92 |
| *p* | **0.027** | **0.022** | 0.057 | **0.013** |
| rs11168100 |  |  |  |  |
| AA | 7731.44±1858.15 | 22.89±3.58 | 62.16±8.59 | 1.99±0.73 |
| AT | 8021.59±1891.83 | 24.60±4.05 | 61.24±9.64 | 2.18±0.80 |
| TT | 9390.5±2025.69 | 23.22±2.96 | 55.64±9.31 | 2.63±0.97 |
| *p* | **0.010** | **0.011** | **0.048** | **0.014** |
| rs353300 |  |  |  |  |
| CC | 8529.15±2094.78 | 23.83±3.63 | 59.96±9.70 | 2.22±0.94 |
| CT | 7794.86±1743.63 | 24.11±3.98 | 61.39±9.17 | 2.11±0.76 |
| TT | 7932.38±2071.91 | 22.71±3.58 | 62.01±9.05 | 2.09±0.74 |
| *p* | 0.128 | 0.106 | 0.571 | 0.684 |

95%CI: 95% confidence interval; OR: odds ratio; SNP: single-nucleotide polymorphism.

*p*-value: Calculated by Pearson χ^2^ test.

Bold type indicates statistical significance (*p* < 0.05).
