# Supplementary material for: Correlation analysis between CARMEN variants and alcohol-induced osteonecrosis of the femoral head in the Chinese population
Source: BMC Musculoskelet Disord. 2020 Aug 15;21:547. doi: 10.1186/s12891-020-03553-2 (PMC7429464; doi:10.1186/s12891-020-03553-2)
Supplement: Supplementary file 4 — Additional file 4 Supplementary Table 4 CARMEN haplotypes frequencies associated with ONFH risk [file 12891_2020_3553_MOESM4_ESM.docx]

Supplementary Table 4 *CARMEN* haplotypes frequencies associated with ONFH risk

|  | SNP | Haplotype | Frequency | | Without adjustment | | With adjustment | |
| --- | --- | --- | --- | --- | --- | --- | --- | --- |
|  |  |  | Case | Control | OR(95%CI) | *p*-value | OR(95%CI) | *p*-value |
| Total | rs13177623\|rs12654195 | AG | 0.268 | 0.304 | 0.84(0.66-1.06) | 0.135 | 0.83(0.65-1.05) | 0.116 |
|  | rs13177623\|rs12654195 | GG | 0.044 | 0.051 | 0.86(0.53-1.41) | 0.553 | 0.88(0.54-1.44) | 0.606 |
|  | rs13177623\|rs12654195 | GT | 0.315 | 0.359 | 0.82(0.66-1.03) | 0.083 | 0.82(0.66-1.02) | 0.077 |
|  | rs11168100\|rs353303\|rs353300 | AGC | 0.395 | 0.400 | 0.98(0.80-1.21) | 0.848 | 0.98(0.79-1.20) | 0.825 |
|  | rs11168100\|rs353303\|rs353300 | AAC | 0.115 | 0.073 | 1.60(1.13-2.27) | **0.008** | 1.62(1.14-2.30) | **0.007** |
|  | rs11168100\|rs353303\|rs353300 | TAT | 0.300 | 0.335 | 0.85(0.68-1.07) | 0.168 | 0.85(0.68-1.06) | 0.153 |
|  | rs11168100\|rs353303\|rs353300 | AAT | 0.180 | 0.186 | 0.97(0.75-1.25) | 0.799 | 0.97(0.75-1.26) | 0.830 |
| ≤45ys | rs13177623\|rs12654195 | AG | 0.249 | 0.313 | 0.74(0.54-1.00) | 0.053 | 0.69(0.50-0.95) | **0.022** |
|  | rs13177623\|rs12654195 | GG | 0.049 | 0.067 | 0.72(0.39-1.32) | 0.283 | 0.69(0.37-1.30) | 0.252 |
|  | rs13177623\|rs12654195 | GT | 0.300 | 0.384 | 0.71(0.53-0.94) | **0.018** | 0.66(0.49-0.89) | **0.007** |
|  | rs11168100\|rs353303 | AG | 0.366 | 0.393 | 0.89(0.67-1.19) | 0.430 | 0.87(0.64-1.16) | 0.338 |
|  | rs11168100\|rs353303 | TA | 0.289 | 0.351 | 0.76(0.57-1.02) | 0.071 | 0.73(0.54-0.99) | **0.045** |
|  | rs11168100\|rs353303 | AA | 0.346 | 0.256 | 1.51(1.12-2.05) | **0.007** | 1.64(1.20-2.25) | **0.002** |
|  | rs353300\|rs353299 | TT | 0.186 | 0.149 | 1.28(0.89-1.84) | 0.181 | 1.33(0.92-1.94) | 0.133 |
|  | rs353300\|rs353299 | CC | 0.506 | 0.473 | 1.13(0.86-1.48) | 0.378 | 1.14(0.86-1.51) | 0.369 |
|  | rs353300\|rs353299 | TC | 0.303 | 0.376 | 0.75(0.56-0.99) | **0.043** | 0.73(0.54-0.97) | **0.032** |
| necrotic sites | rs13177623\|rs12654195 | AG | 0.275 | 0.304 | 0.87(0.68-1.12) | 0.270 | 0.86(0.67-1.11) | 0.241 |
|  | rs13177623\|rs12654195 | GG | 0.045 | 0.051 | 0.89(0.53-1.51) | 0.678 | 0.92(0.54-1.55) | 0.745 |
|  | rs13177623\|rs12654195 | GT | 0.324 | 0.359 | 0.86(0.68-1.09) | 0.210 | 0.86(0.68-1.09) | 0.203 |
|  | rs11168100\|rs353303\|rs353300 | AGC | 0.380 | 0.400 | 0.93(0.74-1.16) | 0.497 | 0.92(0.74-1.15) | 0.477 |
|  | rs11168100\|rs353303\|rs353300 | AAC | 0.122 | 0.073 | 1.70(1.18-2.46) | **0.005** | 1.72(1.19-2.49) | **0.004** |
|  | rs11168100\|rs353303\|rs353300 | TAT | 0.310 | 0.335 | 0.89(0.70-1.14) | 0.358 | 0.89(0.70-1.13) | 0.339 |
|  | rs11168100\|rs353303\|rs353300 | AAT | 0.178 | 0.186 | 0.95(0.72-1.26) | 0.731 | 0.96(0.72-1.27) | 0.760 |

95%CI: 95% confidence interval; OR: odds ratio; SNP: single-nucleotide polymorphism.

*p*-value: Calculated by Pearson χ^2^ test.

Bold type indicates statistical significance (*p* < 0.05).
